# Assessment of Bone Mineral Density in Male Patients with Chronic Obstructive Pulmonary Disease by DXA and Quantitative Computed Tomography

**DOI:** 10.1155/2016/6169721

**Published:** 2016-03-21

**Authors:** George Fountoulis, Theodora Kerenidi, Constantinos Kokkinis, Panagiotis Georgoulias, Paschal Thriskos, Konstantinos Gourgoulianis, Ioannis Fezoulidis, Katerina Vassiou, Marianna Vlychou

**Affiliations:** ^1^Department of Radiology, University Hospital of Larissa, University of Thessaly, School of Medicine, Biopolis, 41110 Larissa, Greece; ^2^Pulmonology Clinic, University Hospital of Larissa, University of Thessaly, School of Medicine, Biopolis, 41110 Larissa, Greece; ^3^Department of Radiology, KAT Hospital, 14561 Athens, Greece; ^4^Department of Nuclear Medicine, University Hospital of Larissa, University of Thessaly, School of Medicine, Biopolis, 41110 Larissa, Greece

## Abstract

The purpose of this study is to identify the prevalence of osteoporosis in male patients with chronic obstructive pulmonary disease (COPD) by dual-energy X-ray absorptiometry (DXA) and quantitative computed tomography (QCT) and to compare the diagnostic abilities of the above methods. Thirty-seven male patients with established COPD were examined with DXA and standard QCT in lumbar spine, including L1, L2, and L3 vertebrae. *T*-scores and bone mineral density values were calculated by DXA and QCT method, respectively. Comparative assessment of the findings was performed and statistical analysis was applied. QCT measurements found more COPD patients with impaired bone mineral density compared to DXA, namely, 13 (35.1%) versus 12 (32.4%) patients with osteopenia and 16 (43.2%) versus 9 (16.2%) patients with osteoporosis (*p* = 0.04). More vertebrae were found with osteoporosis by QCT compared to DXA (*p* = 0.03). The prevalence of osteoporosis among male patients with COPD is increased and DXA may underestimate this risk. QCT measurements have an improved discriminating ability to identify low BMD compared to DXA measurements because QCT is able to overcome diagnostic pitfalls including aortic calcifications and degenerative spinal osteophytes.

## 1. Introduction

Chronic obstructive pulmonary disease (COPD) is a disease characterized by nonreversible airflow obstruction [1]. Although the main symptoms originate from the respiratory system, COPD is considered a systemic disease, and COPD patients have various comorbidities, not the least of which is osteoporosis [2, 3]. A meta-analysis showed that the percentage of COPD patients with osteoporosis is approximately 35% and that osteoporosis' prevalence is higher in women, in patients in a worse stage of COPD, and in patients with a low body-mass index [4]. COPD patients have a greater prevalence of osteoporosis compared with normal subjects; this has been attributed to the systemic use of oral corticosteroids, to the use of tobacco, to systemic inflammation, and to less ability for exercise [5].

The gold standard of measuring bone density is dual-energy X-ray absorptiometry (DXA). Currently, the method is the examination of choice for diagnosis and follow-up of patients with osteoporosis, as proposed by the International Society for Clinical Densitometry, because of its worldwide availability, low radiation dose, and results' reproducibility [6]. However, the technical drawbacks of this method are well acknowledged [7]; as DXA is a two-dimensional method of assessing bone mineral density (BMD), superimposed tissue may cause artifacts and inaccurate measurements.

Quantitative computed tomography (QCT) may serve as an alternative tool for bone densitometry with the advantage that its results are independent of extraspinal pathology, such as aortic calcifications [8–10]. QCT results in the spine have been found to be reproducible, and they are considered a prognostic factor for pathologic fractures [6, 11]. In fact, comparisons between QCT and DXA have shown that the former is better at identifying vertebral fractures [12]. However, the obligatory use of a reference standard and the image postprocessing that is required, in addition to the increased cost and radiation dose, has set back the wide use of the method, in spite of its technical superiority. Also, studies based on DXA measurements have been long validated, whereas studies using QCT are fewer and with less subjects [8]. However, QCT measurements allow volumetric trabecular bone assessment without superimposition of cortical bone and other tissues, rendering the method effective in estimation of bone strength [13].

Our aim in this study was to analyze the prevalence of osteoporosis in male patients with COPD by using either DXA or QCT and to investigate the diagnostic ability of each method in detecting abnormal bone mineral density values in the lumbar spine.

## 2. Materials and Methods

### 2.1. Patient Selection

This was a cross-sectional study performed in a single center. Patients were recruited from the outpatient respiratory clinics of a tertiary hospital. The duration of the investigation was 12 months.

The inclusion criteria for the present study were as follows: all patients were male and had a diagnosis of COPD according to Global Initiative for Chronic Obstructive Pulmonary Disease (GOLD) at least 6 months prior to the QCT and DXA examinations. Seventeen patients have received inhaled steroids for more than 2 years as part of their therapy and twenty patients have not received inhaled steroids. All patients were ex-smokers and belonged to Caucasian race. Exclusion criteria for patient selection were known comorbidities that affect the bone mineral status such as hemiplegia due to stroke, severe diabetes, endocrinopathies, known malignancies, and long standing steroid therapy for systemic and/or autoimmune diseases such as rheumatoid arthritis. None of the patients was under antiosteoporotic medication. All patients gave written informed consent, and the study was approved by the hospital's ethical committee.

### 2.2. DXA Examination

DXA examination in the lumbar spine (L1–L3) was performed in a Hologic Discovery QDR Series Densitometer (Hologic Inc., Bedford, MA). The patients were scanned in the supine position, with their knees held high and bent in a right angle and their calves resting on a cushion, in order to reduce the normal spinal lordosis, as proposed by the manufacturer. The patients were in light clothing during the examination. The device is calibrated daily, according to the manufacturer's instructions for quality control, and has a coefficient of variation of 1.0% for the spine phantom. The examinations were reviewed by a radiologist (GAF), and regions of sclerosis or large osteophytes were excluded from the analysis. BMD was expressed in g/cm^2^, and the patients' *T*-score was estimated using the National Health and Nutrition Examination Survey database, as provided by the manufacturer. The patients' vertebrae were classified according to the WHO classification, and they were classified as normal, if their *T*-score was higher than or at −1.0, as osteopenic, if their *T*-score was between −1.0 and −2.5, and as osteoporotic if the *T*-score was −2.5 or lower.

### 2.3. QCT Examination

QCT examination was performed using a Toshiba (Tokyo, Europe) Aquilion 16-slice computed tomography unit. All patients were scanned in the supine position, with a solid QCT phantom (Mindways Software Inc., Austin, TX, USA) placed below them, on the midline in the thoracolumbar region. The scanning protocol was the same for all patients. Scan parameters were 120 kV, 100 mAs, 1 mm (slice thickness), and 40 cm (field of view (FOV)). According to our quality control, this protocol has an in vitro coefficient of variation of 3.8 ± 2.2% for the phantom we use, which is on par with other researchers [14]. After the scan, images were reviewed in the sagittal plane to identify any loss of vertebral height or wedge deformity, indicating an osteoporotic fracture. Then, 10 mm thick nonangled reconstructions were made through the center of each T12-L3 vertebra, excluding those with osteoporotic fractures and vertebral osseous lesions such as hemangiomas. Trabecular BMD measurement was performed using a software package: QCT PRO 4.2.3. An oval region of interest (ROI) was placed in the trabecular bone at the anterior part of three vertebral bodies (L1–L3), excluding areas of sclerosis, the area of the basivertebral vein, and the vertebral cortex. A vertebral BMD of above 120 mg/cm^3^ of hydroxyapatite was classified as normal, a vertebral BMD within 80–120 mg/cm^3^ was classified as osteopenic, and a BMD below 80 mg/cm^3^ was classified as osteoporotic [8, 15]. The patients did not receive any intravenous or oral contrast media 3 days prior to the study. None of the patients had prior surgery with metal implants.

The time interval between DXA and QCT scan was 14 days ± 6 days and 7 patients performed both studies on the same day.

### 2.4. Statistical Analysis

Chi-square test was used to measure the overall difference between DXA and QCT measurements in detecting osteoporotic, osteopenic, and normal patients and the intraclass differences per lumbar level of the two methods. The cohort of patients was divided into two subgroups based on their GOLD stage, the first with patients classified at GOLD stages I and II and the second with patients at GOLD stages III and IV; chi-square test was performed in order to measure any differences between DXA and QCT regarding overall lumbar vertebrae. Regarding chi-square test, Monte Carlo simulation was used.

Multivariate linear regression was used to estimate the effect of BMI, GOLD stage, age, inhaled steroids, and DXA measurements on QCT measurements. The same analysis was carried out on single QCT site measurements using linear regression as the predicted variable and BMI, GOLD stage, age, inhaled steroids, and single site DXA measurements as the predictors. The sample size of 37 patients with COPD is not a large number; thus it may compromise the statistical significance especially in multivariate analysis.

*p* values less than 0.05 were considered statistically significant. All data were analyzed using R for statistical computing.

## 3. Results

Thirty-seven patients were enrolled in this study, yielding a total number of 111 vertebrae (L1–L3) for the DXA examination and the QCT analysis. The mean age ± standard deviation of our patients was 67.8 ± 7.5 years (age range: 52–86 years old). According to their GOLD stage, based on FEV1, two patients were classified as GOLD stage I, 15 as stage II, 12 as stage III, and 8 as stage IV (Table 1).

According to the DXA measurements, 59 (53.1%) vertebrae had normal BMD, 32 (28.8%) were osteopenic, and 20 (18%) were osteoporotic. According to the QCT measurement, 26 (23.4%) vertebrae had normal BMD, 48 (43.2%) were osteopenic, and 37 (33.3%) were osteoporotic (Table 2). Overall, we found a statistically significant difference in detection of normal vertebrae between DXA and QCT (*p* = 0.0004) and in detection of osteoporotic vertebrae (*p* = 0.03). The classification of our patients into two groups based on their GOLD stage showed that there was a statistically significant difference in detection of normal vertebrae only in the second group (*p* = 0.001), namely, among patients with GOLD stages III and IV (Table 2).

On a per-patient basis, according to the mean L1–L3 DXA measurement, 16 (43.2%) were normal, 12 (32.4%) were osteopenic, and 9 (16.2%) were osteoporotic. According to the mean L1–L3 QCT measurement, 8 patients (21.6%) were normal, 13 (35.1%) were osteopenic, and 16 (43.2%) were osteoporotic (Table 3).

Our analysis showed that DXA and QCT measurements were found to have an overall statistically significant difference (*p* < 0.001) on classifying a patient as osteoporotic, osteopenic, or normal. QCT method detected overall more patients with abnormal BMD values compared to DXA.

On a per-level basis, our analysis showed a statistically significant difference between DXA and QCT measurements of L1, L2, and L3 vertebra. QCT method found, as previously shown, more cases with abnormal bone mineral density values compared to DXA (Table 3). The *p* values for each level were as follows: *p* < 0.01 for L1, *p* < 0.001 for L2, and *p* < 0.01 for L3 vertebra, respectively.

Our multivariate analysis showed that in general QCT measurements seem to be related to the age of the patient (*p* = 0.002) with a combined mean decrease of 17.5 units for every added decade. There is also a joint relationship (interaction) to DXA measurements (*p* < 0.001) and the fact that whether a patient is taking steroids or not. Those measurements follow a different trend within each group of steroid intake (*p* = 0.02). BMI and COPD stages do not seem to have any significant effect on QCT measurements (*p* = 0.2 and *p* = 0.5, resp.). Individual QCT measurement are related to DXA measurements, patient's age, and steroid intake in all cases except L3-QCT site as depicted in Table 3. No steroid-DXA interaction was detected when we considered single site measurements, even though steroid intake was a significant factor for both L1-DXA (*p* = 0.03) and L2-DXA sited (*p* = 0.02) but not for L3-DXA site (*p* = 0.07). Again BMI and COPD stage were not significant (Table 4).

## 4. Discussion

In the present study, we used quantitative computed tomography (QCT) to investigate the prevalence of low BMD in patients with COPD and correlated our findings with DXA measurements, which are considered the gold standard.

The prevalence of low BMD by QCT in our patients was high; 37.8% of our patients were osteopenic and 43.2% were osteoporotic. These results are in accordance with a published meta-analysis, which has shown that, in COPD patients, there is a prevalence of osteoporosis of 35.1% and a prevalence of osteopenia of 38.4% [4]. Most of the studies included in the meta-analysis used DXA measurements; only R Core Team [16] utilized quantitative heel ultrasound and Dimai et al. [17] used peripheral QCT in the forearm to classify their patients. A recent publication by Jaramillo et al. [18] confirms our findings that COPD and smoking are combined risk factors that may influence negatively the bone mineral density of male patients and QCT has been proposed as the screening method of choice in order to identify better such patients with impaired bone mineral density.

**Figure 1:**
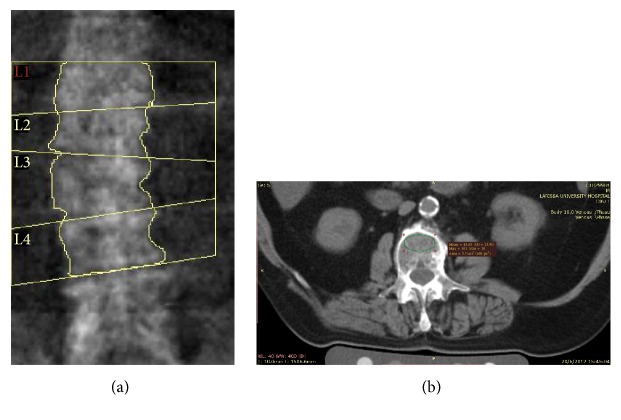
(a) DXA image of a lumbar spine with mild scoliosis in a male patient with COPD. DXA measurement of the L2 vertebra was consistent with normal BMD (1.054 g/cm^2^, *T*-score −0.4). (b) QCT measurement of L2 vertebra (56.3 mg/cm^3^) was consistent with osteoporosis. The patient also had a vertebral wedge deformity at T12. Notable abdominal aortic calcifications are noted in axial CT image that may have contributed to the discrepancy of the results.

We found significant differences in the classification of separate vertebrae by means of QCT and DXA, with the QCT measurements indicating higher percentages of both osteopenia and osteoporosis. Reports have shown that DXA measurements could be normal in the presence of osteoporotic fractures [17, 19–21]. Reasons that have been offered for the discrepancy of the results are the technical limitations of DXA as a method, which is unable to identify sclerotic areas of degenerative etiology, or abdominal aorta calcifications (Figures 1(a) and 1(b)).

Our findings are in agreement with published data by Li et al. [22] which compared DXA and QCT measurements among postmenopausal women and found that QCT can overcome the abovementioned projecting errors that may lead to DXA erroneous diagnosis in the area of lumbar spine. Another study by Liu et al. [23] also concluded that QCT measurements were more valuable in estimating bone loss in the lumbar spine among patients with spinal cord injury, compared to the DXA.

Direct comparison of DXA and QCT has been made, and QCT has been found to be more accurate at predicting fractures, albeit at the cost of increased radiation dose and financial cost [12, 20, 21]. The classification of our patients in two groups according the GOLD stage showed that there was a statistically significant difference in detection of normal vertebrae between DXA and QCT only among patients with advance disease, whereas in the overall group we found statistically significant differences in detection of both normal and osteoporotic vertebrae. Our data suggested that some patients with COPD could suffer from osteoporosis even if their DXA measurements are within normal limits, and thus, further investigation with QCT could be warranted, especially in those with low body weight or advanced disease [4].

Based on our findings, the stages of COPD and BMI are not correlated with higher prevalence of osteoporosis, although these factors pose a controversial issue in the literature. The TORCH study [24] demonstrated a higher prevalence of osteoporosis and osteopenia at baseline, in those patients with spirometrically confirmed COPD, but there was no association between FEV1 impairment and BMD when adjusted by age and gender. Other studies, however, support that there is a positive correlation between stage of COPD and osteoporosis which is statistically significant [25]. Larger numbers of patients need to be examined in order to clarify further the above relationship. The hypothesis that male patients are influenced mainly by senile osteoporosis and therefore the skeletal transition is more gradual although other comorbidities may exist should be tested as well.

There is a statistically significant relationship between steroids and osteoporosis and between age and osteoporosis as shown in Table 4. The effect of steroids is well established in the literature [24, 25] and it may be suggested that, even in low dose schemes, there is an increased risk of fractures among patients with COPD due to low BMD.

Our study has certain limitations. First, our sample population was small, from a single center. However, on a per-vertebra analysis, we found that there was a statistical difference between DXA and QCT measurements, with the latter being lower. The statistical analysis did not include correlations with DXA measurements of the hip, because this was beyond the purpose of the present study. Additionally, our QCT reconstructions were not angled parallel to the vertebral end-plates, yet previous research has shown that as long as areas of sclerosis are ignored, the size and shape of the area of measurement have a high correlation factor [22]. Besides, our purpose was to produce a practical way of estimating BMD in our patients, and reconstructing oblique images from the original data is time consuming. A further limitation was that we have not used Trabecular Bone Score (TBS) software since it was not available in our unit.

Based on our findings, it may be suggested that the prevalence of low BMD in patients with COPD was higher by using QCT compared to DXA, a fact that could be attributed to aortic calcifications and other artifacts at DXA. However, dedicated QCT studies for the detection of alterations in bone mineral density in large sample of patients increase the overall diagnostic costs and radiation exposure. Further studies need to be performed after proper selection of patients in order to explore the full potential of the above methodology and its role in the management of patients with COPD.

## Figures and Tables

**Table 1:** Demographics of patients.

Number of patients	37

Age	67.7 ± 7.5^†^

Height	1.68 ± 0.07^†^

Weigh	77.2 ± 13.9^†^

BMI	27.2 ± 4.6^†^

COPD	
Stage I	2
Stage II	15
Stage III	12
Stage IV	8

Steroids	
Yes	20
No	17

^†^Mean ± standard deviation.

**(a):** 

		Normal	Osteopenic	Osteoporotic
Vertebrae (*N* = 111)	DXA	59 (53.1%)	32 (28.8%)	20 (18%)
	QCT	26 (23.4%)	48 (43.2%)	37 (33.3%)

	*p* value	**0.0004**	0.09	**0.03**

Patients (*N* = 37)	DXA	16 (43.2%)	12 (32.4%)	9 (16.2%)
	QCT	8 (21.6%)	13 (35.1%)	16 (43.2%)

	*p* value	0.15	0.69	**0.04**

**(b):** 

Vertebrae		Normal	Osteopenic	Osteoporotic
Stage GOLD I + II (*N* = 51)	DXA	28 (54.9%)	18 (35.3%)	5 (9.8%)
	QCT	16 (31.3%)	22 (43.1%)	13 (25.4%)

	*p *value	0.09	0.6	0.09

Stage GOLD III + IV (*N* = 60)	DXA	31 (51.6%)	14 (23.3%)	15 (25%)
	QCT	10 (16.6%)	26 (43.3%)	24 (40%)

	*p *value	**0.001**	0.08	0.2

^**∗**^Dual X-ray absorptiometry.

^**∗∗**^Quantitative computed tomography.

**Table 3:** DXA^*∗*^ and QCT^*∗∗*^ detection rates.

	QCT		DXA	
	Normal	Diminished^†^	Normal	Diminished^†^
Measurement levels				
L1	9	28	16	21
L2	8	29	22	15
L3	9	28	21	16

Patients	8	29	16	21

^†^Osteoporotic and osteopenic

^**∗**^Dual X-ray absorptiometry

^**∗∗**^Quantitative computed tomography.

**Table 4:** Correlation between QCT and DXA measurements with respect to age and steroid therapy.

QCT measurements	DXA measurements			Age	Steroid
	L1DXA	L2DXA	L3DXA		
L1QCT	<0.001	—	—	0.001	0.03
L2QCT	—	<0.001	—	<0.001	0.02
L3QCT	—	—	<0.001	0.002	0.07^†^

^†^ *p* value > 0.05.
