# Corrigendum to “Assessment of Bone Mineral Density in Male Patients with Chronic Obstructive Pulmonary Disease by DXA and Quantitative Computed Tomography”

**DOI:** 10.1155/2018/2165342

**Published:** 2018-05-02

**Authors:** George Fountoulis, Theodora Kerenidi, Constantinos Kokkinis, Panagiotis Georgoulias, Paschal Thriskos, Konstantinos Gourgoulianis, Ioannis Fezoulidis, Katerina Vassiou, Marianna Vlychou

**Affiliations:** ^1^Department of Radiology, University Hospital of Larissa, University of Thessaly, School of Medicine, Biopolis, 41110 Larissa, Greece; ^2^Pulmonology Clinic, University Hospital of Larissa, University of Thessaly, School of Medicine, Biopolis, 41110 Larissa, Greece; ^3^Department of Radiology, KAT Hospital, 14561 Athens, Greece; ^4^Department of Nuclear Medicine, University Hospital of Larissa, University of Thessaly, School of Medicine, Biopolis, 41110 Larissa, Greece

In the article titled “Assessment of Bone Mineral Density in Male Patients with Chronic Obstructive Pulmonary Disease by DXA and Quantitative Computed Tomography” [1], there were errors in Table 2(a) concerning the *p* values calculated for the osteopenic and osteoporotic patients, as raised in a letter to the editor by Moran and Zamorano [2].

Based on the comments raised in the letter [2], the authors redid the analysis. Accordingly, the following sections should be corrected:

In Abstract, “Comparative assessment of the findings was performed and statistical analysis was applied. QCT measurements found more COPD patients with impaired bone mineral density compared to DXA, namely, 13 (35.1%) versus 12 (32.4%) patients with osteopenia and 16 (43.2%) versus 9 (16.2%) patients with osteoporosis (*p* = 0.04). More vertebrae were found with osteoporosis by QCT compared to DXA (*p* = 0.03)” should be corrected to “Cohen kappa statistic showed that overall the agreement between the two methods was moderated (0.45). The GEE (generalized estimating equation) analysis showed that the probability of detecting abnormal bone mineral density was three times greater when using QCT compared to DXA measurements.”

In Statistical Analysis, “Chi-square test was used to measure the overall difference between DXA and QCT measurements in detecting osteoporotic, osteopenic, and normal patients and the intraclass differences per lumbar level of the two methods. The cohort of patients was divided into two subgroups based on their GOLD stage, the first with patients classified at GOLD stages I and II and the second with patients at GOLD stages III and IV; chi-square test was performed in order to measure any differences between DXA and QCT regarding overall lumbar vertebrae. Regarding chi-square test, Monte Carlo simulation was used” should be corrected to “The Cohen kappa statistic was used to access whether QCT and DXA agree or differ in their ability to detect normal, osteopenic, and osteoporotic patients. Values less than 0.4 indicate poor agreement, and values more than 0.75 indicate excellent agreement. In-between values indicate moderate agreement.

In order to compare the diagnostic ability of the two methods between normal and diminished patients (i.e., osteopenic and osteoporotic), we performed the McNemar test to explore if age, steroids, and the diagnostic method used are significant factors to a patient's classification as normal or diminished, the GEE (generalized estimating equation) procedure was employed.”

In Results, “Overall, we found a statistically significant difference in detection of normal vertebrae between DXA and QCT (*p* = 0.0004) and in detection of osteoporotic vertebrae (*p* = 0.03). The classification of our patients into two groups based on their GOLD stage showed that there was a statistically significant difference in detection of normal vertebrae only in the second group (*p* = 0.001), namely, among patients with GOLD stages III and IV (Table 2).

On a per-patient basis, according to the mean L1–L3 DXA measurement, 16 (43.2%) were normal, 12 (32.4%) were osteopenic, and 9 (16.2%) were osteoporotic. According to the mean L1–L3 QCT measurement, 8 patients (21.6%) were normal, 13 (35.1%) were osteopenic, and 16 (43.2%) were osteoporotic (Table 3).

Our analysis showed that DXA and QCT measurements were found to have an overall statistically significant difference (*p* < 0.001) on classifying a patient as osteoporotic, osteopenic, or normal. QCT method detected overall more patients with abnormal BMD values compared to DXA.

On a per-level basis, our analysis showed a statistically significant difference between DXA and QCT measurements of L1, L2, and L3 vertebra. QCT method found, as previously shown, more cases with abnormal bone mineral density values compared to DXA (Table 3). The *p* values for each level were as follows: *p* < 0.01 for L1, *p* < 0.001 for L2, and *p* < 0.01 for L3 vertebra, respectively.” should be changed to “Overall, the Cohen Kappa statistic showed a moderate-to-poor agreement 0.45 (0.24, 0.66) between the two diagnostic methods. The comparative per-level analysis showed that the agreement was even poorer (Table 2). The McNemar test further showed that the detecting ability of the two methods is not the same (*p* = 0.013). The distribution of normal, osteopenic, and osteoporotic vertebrae is shown in Table 3.”

Also, the following paragraph should be added at the end of the section:

“When we investigated the relation of DXA and QCT with age, steroid intake and the fact that the patient had normal or diminished bone mineral density, we found that age (*p* = 0.23) and steroid intake (*p* = 0.94) were not statistically significant, but the diagnostic method was (*p* = 0.04). The probability of detecting an abnormal vertebra was found to be three times greater by using the QCT method compared to DXA.”

Table 2 should be corrected as follows:

## Figures and Tables

**Table 2:** DXA versus QCT Cohen kappa statistics, per level and overall.

Vertebrae	
L1	0.31 (0.075, 0.55) poor agreement
L2	0.39 (0.19, 0.59) poor agreement
L3	0.42 (0.18, 0.65) moderate agreement
Overall (L1–L3)	0.45 (0.24, 0.66) moderate agreement

DXA: dual X-ray absorptiometry; QCT: quantitative computed tomography.
